# Optimization of the Preparation of Fish Protein Anti-Obesity Hydrolysates Using Response Surface Methodology

**DOI:** 10.3390/ijms14023124

**Published:** 2013-02-01

**Authors:** Liyuan Liu, Yanping Wang, Chen Peng, Jinju Wang

**Affiliations:** 1 Key Laboratory of Food Nutrition and Safety, Ministry of Education, Tianjin University of Science & Technology, Tianjin 300457, China; E-Mails: liuliyuan0206@163.com (L.L.); pengchen1211@mail.tust.cn (C.P.); bioorange@163.com (J.W.); 2 Ocean College of Hebei Agricultural University, Qinhuangdao 066000, China

**Keywords:** anti-obesity, fish protein, kinetics, porcine pancreas lipase, α-amylase, response surface methodology (RSM)

## Abstract

The enzymatic condition for producing the anti-obesity hydrolysates from fish water-soluble protein was optimized with the aid of response surface methodology, which also derived a statistical model for experimental validation. Compared with neutral protease, papain and protamex, the porcine pancreas lipase inhibitory rate of hydrolysates from fish water-soluble protein was higher with alkaline protease. Results showed that the model terms were significant, the terms of lack of fit were not significant, and the optimal conditions for the hydrolysis by alkaline protease were initial pH 11, temperature 39 °C, enzyme dosage 122 U/mL and 10 h of hydrolysis time. Under these conditions, the porcine pancreas lipase and the α-amylase inhibitory rate could reach 53.04% ± 1.32% and 20.03 ± 0.89%, while predicted value were 54.63% ± 1.75%, 21.22% ± 0.70%, respectively. In addition, Lineweaver-Burk plots showed noncompetitive inhibition. The *K*_i_ value calculated was 84.13 mg/mL. These results demonstrated that fish water-soluble protein could be used for obtaining anti-obesity hydrolysates.

## 1. Introduction

With the development of modern society, prevalence of obesity is seriously threatening global health owing to people’s life style of decreased physical activity and intake of high energy food [1,2]. It is well-known that obesity is associated with many chronic diseases such as hyperlipidemia, hypertension, coronary heart disease, type II diabetes, cancers and other serious diseases [3]. Obesity is a disarray of energy balance and primarily considered as a disorder of lipid metabolism. A growing number of enzymes involved in lipid metabolic pathways are being identified and characterized. They represent a rich pool of potential therapeutic targets for obesity. In the course of the lipid metabolism, pancreatic lipase is the key enzyme for the digestion of triglycerides. Dietary triglycerides are hydrolyzed by pancreatic lipase to monoglycerides, free fatty acids and other small molecules, which are absorbed in the intestine and then resynthesize triglycerides for the person of food intake leading to obesity ultimately. So, the inhibition of lipase is efficient to prevent obesity, which is one of the most important methods for valuing anti-obesity activity *in vitro* [4]. Many drugs have been found to treat obesity, such as Orlistat, which is well known as a gastric and pancreatic lipase inhibitor [5]. Another important approach for controlling weight is to reduce or slow dietary carbohydrate digestion and absorption. Starch digestion primarily occurs through the action of α-amylase, yielding both linear maltose and branched isomaltose oligosaccharides, which are further hydrolyzed by α-glucosidases to release absorbable monosaccharide [6,7]. By inhibiting α-amylase involved in carbohydrate digestion, it significantly delays postprandial hyperglycemia and hinders energy production [8]. Therefore, in this study, the inhibition of pancreatic lipase, assisted with α-amylase inhibitory activity, was selected as the evaluation criterion of anti-obesity.

In several studies, it has been reported that the anti-obesity activities were shown by protamine [9], wheat proteins [10,11], soybean cotyledon proteins [12], defatted rice bran proteins and lupin proteins [13,14], as well as soybean hydrolysates [15], black soybean hydrolysates [16,17], bovine hemoglobin hydrolysates [18]. Only a few studies have reported the hypolipidemic, cholesterol-lowering [19], antihypertensive [20], antioxidant effects [21–24], immunomodulating [25,26] and antiproliferative activities of fish protein and hydrolysates. There have hitherto been no reports on the inhibitory effects of fish protein hydrolysates (FPH) on obesity. Enzymatic proteolysis applied to proteinaceous fish by-products has been described as a means to transform these materials into more marketable and value-added products with improved functional and biological properties.

Response surface methodology (RSM) is an effective tool for optimizing reaction parameters. Many bioactive peptides or hydrolysates have been optimized using RSM, including the angiotensin-converting enzyme inhibitors from whey protein and collagen, enzymatic hydrolysis of protein concentrates or waste protein by commercial proteases [27–30], and antioxidant hydrolysates from shrimp processing discards and saithe [31].

In this study, RSM was used to identify the most important variables for optimizing the enzymatic hydrolysis conditions, under which it was expected to obtain original and effective hydrolysates for anti-obesity, which might be an excellent alternative strategy for the development of safe and effective anti-obesity drugs.

## 2. Results and Discussion

### 2.1. Effects of Different Proteases Hydrolysis for Producing Anti-Obesity Hydrolysates

**Figure 1:**
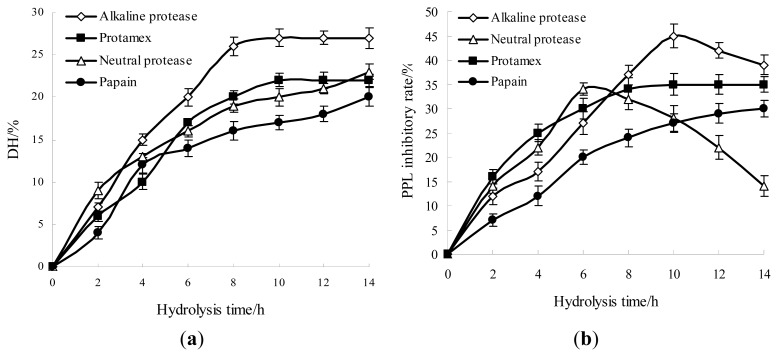
Degree of hydrolysis (**a**) and porcine pancreas lipase (PPL) inhibitory rate (**b**) during different stages of enzymatic hydrolysis using four proteases. The error bars represent standard deviations from three independent samples.

Figure 1a shows the hydrolysis curves of each protease obtained at the optimal initial pH and temperature, with the substrate concentration of 0.22% (*w*/*v*) fish water-soluble protein and enzyme dosage 100 U/mL. The degree of hydrolysis (DH) increased significantly when the hydrolysis time was prolonged from 0 to 8 h, and peaked at 10 h. When hydrolysis time was longer than 10 h, DH started to maintain a dynamic equilibrium with a slight increase. These results indicated that four proteases showed certain catalytic activities. During the hydrolysis processing, the DH value of alkaline protease was significantly higher than that of other proteases.

Suitable enzymes for marine protein hydrolysis should be selected according to various criteria, such as flavor extraction, physicochemical properties of the resulting hydrolysate, or reduction of bitterness by limiting the release of hydrophobic amino acids. From Figure 1b, a comparative study of four proteases was performed on PPL inhibitory activity of FPH. The highest PPL inhibitory rate was obtained using alkaline protease at the maximal point compared to neutral protease, protamex and papain. Alkaline protease has been chosen by many authors due to its efficiency to hydrolyze fish protein at laboratory scale [32,33]. Klompong *et al*. [23] have reported at low DH (5%), protein hydrolysate of yellow stripe trevally (*Selaroides leptolepis*) with alkaline protease exhibited a better DPPH radical-scavenging activity compared to Flavourzyme. During the reaction process, the PPL inhibitory rate increased at the beginning, reached the maximal value, and then decreased rapidly, when the FPH were hydrolyzed by neutral protease. Similarly, the PPL inhibitory rate of FPH with alkaline protease increased with reaction time, and reached the maximal value at 10 h, and then decreased slowly. This indicated that a hydrolysis time of 10 h was fit to obtain anti-obesity hydrolysates. A very long hydrolysis time might induce the degradation of polypeptide, which would lead to changes in the polypeptide molecular weight and a decrease in PPL inhibitory rate. Nevertheless, the structure-activity relationship and the anti-obesity mechanism of active peptides were not yet fully elucidated. The anti-obesity activity of hydrolysates has been related both to the enzyme specificity, the degree of hydrolysis, and the amino acid composition and sequence of the different peptides released. Therefore, it can be concluded that alkaline protease could be a suitable protease for catalytic hydrolysis of fish water-soluble protein, and 10 h was selected as the center point of the hydrolysis time in the RSM experiment.

### 2.2. Fitting the Model

RSM was used to develop a predicted model for optimizing the PPL and α-amylase inhibitory activities of FPH. The experimental conditions and the corresponding values from the experimental design were presented in Table 1. All tests were performed in triplicate. Values represented the mean ± standard deviations.

Table 2 showed the ANOVA for the PPL and the α-amylase inhibitory activities of FPH. It was found that the two statistical model were significant at a 99% confidence level (*p* < 0.01), which meant there was only a 0.01% chance that a “model *F*-value” could occur due to noise. As the test of lack of fit hypothesis was not significant (*p* > 0.05) in model equations, the models were fitted to the PPL and the α-amylase inhibitory rate data.

Values of *p* < 0.05 indicated that the model terms were significant and the models had some power to explain the variation in the responses. In this case, for the PPL inhibitory activity, *χ*_1_, *χ*_2_, *χ*_3_, *χ*_4_, *χ*_1_*χ*_3_, *χ*_2_*χ*_4_, *χ*_3_*χ*_4_ were significant model terms. The higher the F value, the more important was the role in the hydrolysis process; therefore, enzyme dosage (*χ*_3_) played a dominant role in the process. Ren *et al*. [34] have reported that [E]/[S] exerted the significant effect on antioxidant activity of the hydrolysates derived from grass carp sarcoplasmic protein. Zhuang *et al*. [35] have also found [E]/[S] exhibited the highest significant effect on hydroxyl radical scavenging activity of jellyfish umbrella collagen hydrolysates. Furthermore, the interactive terms (*χ*_3_*χ*_4_) presented a significant effect on the PPL inhibitory rate, namely, the interactions between enzyme dosage and hydrolysis time.

For the PPL inhibitory activity, the high coefficient of determination value (*R*^2^ = 0.9800) indicated that 98.00% of the variability in the response could be explained by the model. The adjusted *R*^2^ value and predicted *R*^2^ value for responses were 0.9599 and 0.8908 respectively, which also presented a model of a good fit. Therefore, the proposed models were adequate for presenting the real relationship among the parameters chosen.

The best explanatory model equations for the PPL inhibitory rate (Y_1_) and the α-amylase inhibitory rate (Y_2_) of FPH were as follows:

(1) $$\begin{array}{c} {\text{Y}}_{1}=53.39-2.84\chi_{1}-4.24\chi_{2}+5.24\chi_{3}+1.99\chi_{4}-1.35\chi_{1}\chi_{2}+2.00\chi_{1}\chi_{3}+1.72\chi_{1}\chi_{4}+\\ 1.32\chi_{2}\chi_{3}-2.25\chi_{2}\chi_{4}-4.86\chi_{3}\chi_{4}-7.95{\chi_{1}}^{2}-8.96{\chi_{2}}^{2}-5.49{\chi_{3}}^{2}-10.80{\chi_{4}}^{2} \end{array}$$

(2) $$\begin{array}{c} {\text{Y}}_{2}=20.44+1.16\chi_{1}+0.70\chi_{2}+2.24\chi_{3}+1.25\chi_{4}-1.20\chi_{1}\chi_{2}-0.080\chi_{1}\chi_{3}-0.98\chi_{1}\chi_{4}-\\ 0.39\chi_{2}\chi_{3}-1.29\chi_{2}\chi_{4}-0.44\chi_{3}\chi_{4}-1.49{\chi_{1}}^{2}-1.88{\chi_{2}}^{2}-1.74{\chi_{3}}^{2}-3.35{\chi_{4}}^{2} \end{array}$$

### 2.3. Analysis of Response Surfaces

**Figure 2:**
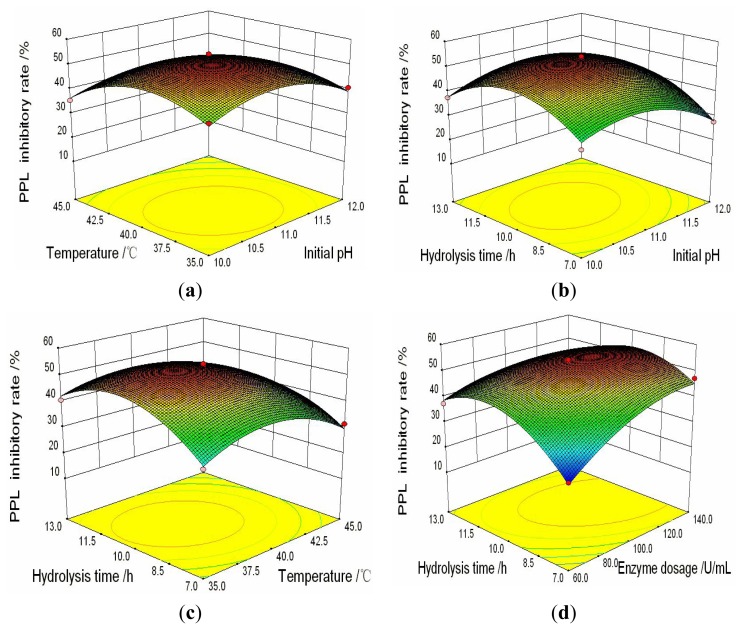
Response surface plots and contour plots for the interactive effects of variables on the porcine pancreas lipase (PPL) inhibitory rate. (**a**) the interactive effects of initial pH and temperature, maintaining fixed enzyme dosage 100 U/mL, hydrolysis time 10 h; (**b**) the interactive effects of initial pH and hydrolysis time, maintaining fixed enzyme dosage 100 U/mL, temperature 45 °C; (**c**) the interactive effects of temperature and hydrolysis time, maintaining fixed enzyme dosage 100 U/mL, initial pH 11; (**d**) the interactive effects of enzyme dosage and hydrolysis time, maintaining fixed initial pH 11, temperature 45 °C.

**Figure 3:**
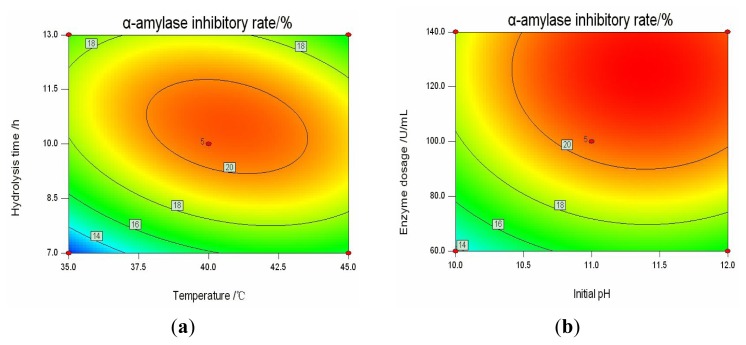
Contour plots for the interactive effects of variables on the α-amylase inhibitory rate. (**a**) the interactive effects of hydrolysis time and temperature, maintaining fixed enzyme dosage 100 U/mL, initial pH 11; (**b**) the interactive effects of enzyme dosage and initial pH, maintaining fixed hydrolysis time 10 h, temperature 45 °C.

Three dimensional plots of the responses based on Equations 1 and 2 were presented in Figure 2 and Figure 3 respectively, where two variables were kept at central point levels and the other two were allowed to vary within the experimental range.

As can be seen from Figure 2a, at a fixed temperature, the PPL inhibitory rate increased at the beginning and then decreased with increase of initial pH value. When the hydrolysis temperature was increased from 35 to 40 °C, there was an increasing trend in the PPL inhibitory rate of FPH, and the maximum inhibitory rate was observed at about 40 °C. The inhibitory rate decreased gradually, when the hydrolysis temperature was higher than 40 °C. The report by Hou *et al*. found that the similar effect of temperature on reducing power of soybean hydrolysates [36]. Guerard *et al*. have also reported during the hydrolysis of shrimp processing discards, temperature and pH played the similar impact on antiradical activity [31]. There were several reasons for these changes in inhibitory rate: firstly, the temperature in our study covered with the range of 35–45 °C, some proteins in the hydrolysates might be denatured at their denaturation temperature. Meanwhile, the activity of alkaline protease might be decreased. At lower temperatures, the rate of enzyme heat-inactivation was slower in comparison with the rate of the enzyme catalyzed reaction. At higher temperatures, the increased heat-inactivation rate led to a faster decrease in the number of active catalyst molecules [37]. In addition, high temperature also increased the cost of the hydrolysis process.

The variation of the PPL inhibitory rate with changing initial pH and hydrolysis time were presented in Figure 2b. Initial pH showed a quadratic effect on the response, hence the PPL inhibitory rate firstly increased and then decreased with initial pH increasing. The report by Cao *et al*. [38] also showed the similar effect of pH on autolysis processing of shrimp head. The solubility of protein was affected by pH value, because of the isoelectric point of each protein. In addition, the pH of the hydrolysis condition decreased with the reaction time. In order to keep the pH at optimal condition as long as possible, without influencing the activity of protease, it was suggested that, when the initial pH was given at about 11, alkaline protease exhibited the highest activity.

It was clear from Figure 2c that at the designed range of temperature from 35 to 45 °C, the PPL inhibitory rate increased quickly at the start and then decreased slowly with the time increasing. Reaction time had a positive linear effect on the inhibitory rate. However, further an increase in reaction time resulted in a little decrease in the inhibitory rate. It was in accordance with the previous study by Song *et al*. [39], which reported the hydrolysis time influenced the antibacterial activity of Half-Fin Anchovy (*Setipinna taty*) hydrolysates in a liner manner at lower levels of pH, while at upper levels of pH, the influence was quadratic. This phenomenon could be probably explained as: The degradation of fish water-soluble protein was mainly a chemical process achieved by hydrolysis of peptide bond. The number of hydrolyzed peptide bonds increased with the extension of the reaction time, and the inhibitory rate increased. While the reaction time was further extended, the decrease in the inhibitory rate became due to the further hydrolysis of the functional polypeptides, and the polypeptides activity declined.

Figure 2d illustrated the effects of the enzyme dosage and the hydrolysis time on the PPL inhibitory rate while maintaining the temperature at 40 °C and the initial pH at 11. It can be found that the enzyme dosage had a significant positive linear effect on the PPL inhibitory rate when the enzyme dosage was lower than 100 U/mL. For a dosage over this critical value, little enhancements in the PPL inhibitory rate attributable to the increase of enzyme dosage were observed, which could be explained by the fact that the enzyme had saturated the hydrolysates. Song *et al*. [39] have also reported the similar effect of enzyme dosage on the antibacterial activity of Half-Fin Anchovy (*Setipinna taty*) hydrolysates.

The effects of variables on α-amylase inhibitory activity were similar to those on pancreatic lipase inhibitory activity. It can be concluded from Table 1, considering the interactive effects of variables on the α-amylase inhibitory rate, that *χ*_1_*χ*_2_, *χ*_1_*χ*_4_, *χ*_2_*χ*_4_ were significant model terms, while *χ*_1_*χ*_3_, *χ*_2_*χ*_3_, *χ*_3_*χ*_4_ were not significant model terms. Figure 3 exhibited the interactive effects of temperature (*χ*_2_) and hydrolysis time (*χ*_4_) on the α-amylase inhibitory rate, as well as the effects of initial pH (*χ*_1_) and enzyme dosage (*χ*_3_). 2D contour plots showed the interrelationships between two tested variables and the relationship between responses and experimental levels of each variable. Different shapes of contour plots indicated different interactions between two variables. A full elliptic contour was observed in Figure 3a, indicating a significant interaction between temperature and hydrolysis time. In the contrast, circular contour plot, which signaled non-significance for the interactions between initial pH and enzyme dosage, was clearly shown in Figure 3b.

### 2.4. Optimization of the Hydrolysis Conditions and Validation of Model

The optimum values were found by solving the regression equation analytically. The optimal hydrolysis reaction conditions were calculated as follows: initial pH 11.08, temperature 39.50 °C, enzyme dosage 121.76 U/mL and hydrolysis time 10.21 h. Considering the cost and the operating convenience of the hydrolysis process, the optimal values of variables were determined as follows: initial pH 11, temperature 39 °C, enzyme dosage 122 U/mL and time 10 h. To validate the predicted model, the tests were carried out at optimal conditions for pH, temperature, enzyme dosage, and hydrolysis time in triplicate. The predicted response Y_1_ (54.63% ± 1.75%) and Y_2_ (21.22% ± 0.70%) were experimentally verified (53.04% ± 1.32%, 20.03% ± 0.89%, *n* = 3).

**Figure 4:**
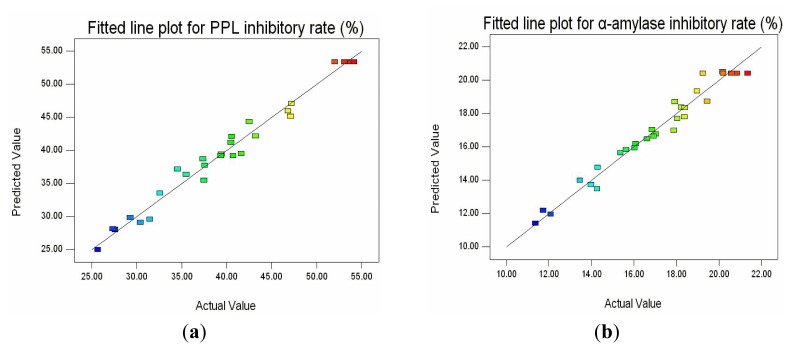
Fitted line plot indicating the closeness between predicted values and experimental values for porcine pancreas lipase (PPL) inhibitory rate (**a**) and α-amylase inhibitory rate (**b**).

The adequacy of the response surface equations was indicated by a comparison between the experimental value and the predicted data. The comparison was done by generating a fitted-line plot for the results obtained, showing how close it was to or how far it deviated from the fitted line. As shown in Figure 4a,b, the agreement between predicted values and experimental values confirmed that the response surface models were adequate for predicting the varied enzymatic properties as functions of the conditions.

### 2.5. Manner of Inhibition by FPH

**Figure 5:**
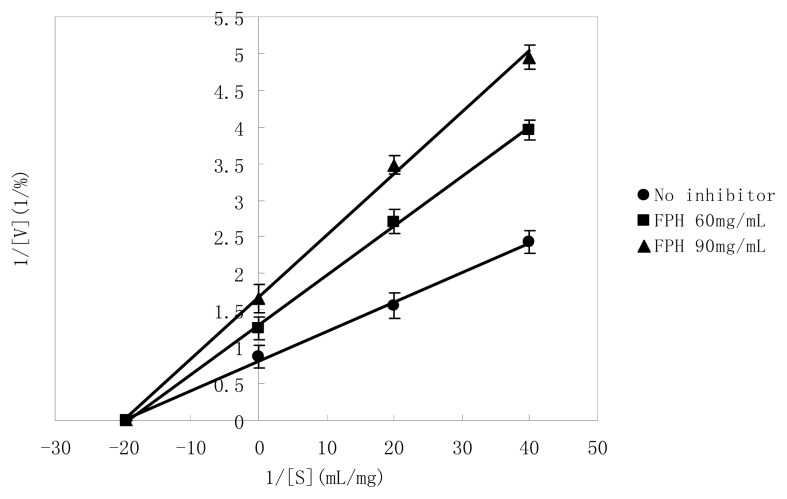
Lineweaver-Burk plots for the inhibition of porcine pancreas lipase (PPL) by fish protein hydrolysates (FPH). The reactions were performed at a constant concentration [0 mg/mL (●), 60 mg/mL (■) and 90 mg/mL (▲)] of polypeptide in hydrolysates. Each bar showed the standard deviations. Values were the means of triplicate analyses.

The inhibitory mechanism of FPH was shown in Figure 5. In order to clarify the kinetics of inhibition against PPL by FPH, the inhibitory type was determined by Lineweaver-Burk plots, and kinetic parameters (*V*_max_, *K*_m_ and *K*_i_) were calculated by Michealis equation. The inhibitory activity was measured by changing the concentration of the substrate at a constant concentration [0 mg/mL (●), 60 mg/mL (■) and 90 mg/mL (▲)] of polypeptide in hydrolysates. Because the plots obtained by changing the substrate concentration intersected with the *χ* axis, it was indicated that the mechanism of PPL inhibition by FPH was noncompetitive type, which meant that FPH could combine with an enzyme molecule to produce a dead-end complex, regardless of whether a substrate molecule was bound or not. Hence, FPH must bind at a different site from the substrate. Therefore, FPH acted as a PPL inhibitor by forming enzyme-substrate-inhibitor and enzyme-inhibitor complexes to reduce the efficiency of catalysis during the reaction. The similar results were also observed by Li *et al*. [40], which reported the mechanism of pancreatic lipase inhibition by flavonoids was noncompetitive. On the basis of linear regression analysis of Lineweaver–Burk plots and Michealis equation, *V*_max_, *K*_m_ and *K*_i_ were calculated to be 1.237%, 0.0512 mg/mL and 84.13 mg/mL, respectively.

## 3. Experimental Section

### 3.1. Materials and Methods

#### 3.1.1. Materials

Chemicals and enzymes used for this study were listed along with their sources as follows: porcine pancreas lipase (30 U/mg, Glycerol trioleate was used as substrate) was purchased from Jianglai Bioengineering Co. Ltd (Shanghai, China); alkaline protease (200 U/mg, Casein was used as substrate), neutral protease (200 U/mg, Casein was used as substrate), papain (100 U/mg, Casein was used as substrate), and protamex (300 U/mg, Casein was used as substrate) from Noao Bioengineering Co. Ltd (Tianjin, China); crucian carp (*Carassius carassius*) was purchased from market (Tianjin, China). All other chemicals were of reagent grade.

#### 3.1.2. Pretreatment and Enzymatic Hydrolysis

Fish muscle from crucian carp (*Carassius carassius*) was ground to uniformity with a homogenizer after removal of head and viscera. Based on the preliminary experiment, deionized water was added at a water/fish muscle ratio of 10:1 (*v*/*w*) in a conical flask (250 mL). After stirred at 150 rmp for 3 h at 43 °C, the mixture was then cooled and centrifuged at 2000× *g* for 15 min, to collect the supernatant. Fish water-soluble protein in the supernatant was measured by the Biuret method according to Layne (1957) using bovine serum albumin as the standard.

The supernatant was adjusted to a suitable pH with 2.0 M HCl or 2.0 M NaOH, then separately digested by neutral protease, alkaline protease, papain, and protamex (100 U/mL) for 14 h. The hydrolysis temperature was controlled at 45 °C for neutral protease and alkaline protease, 55 °C for papain and protamex. The hydrolysis initial pH was 7.0 for neutral protease, protamex and papain, 10.0 for alkaline protease. The reaction was stopped by boiling for 15 min. Then the supernatant was used for determination of the PPL and the α-amylase inhibitory activities. The optimal protease was chosen according to the DH and the PPL inhibitory rate.

#### 3.1.3. Assay for PPL Inhibitory Activity

The ability of the compounds to inhibit PPL was measured using the method previously reported by Kim *et al*. [41], with some modifications.

Lipase activity was determined by measuring the fatty acid released from olive oil. Briefly, a mixture of olive oil and polyvinyl alcohol (1:3, *v*/*v*) was used as the substrate. Firstly 2.0 mL of the substrate and 2.5 mL of phosphate buffer (0.05 M, pH 7.0) were pre-incubated for 5 min at 40 °C, then 0.5 mL of sample and 0.5 mL of porcine pancreatic lipase solution were added and the enzymatic reactions were allowed to proceed for 15 min at 40 °C. After incubation, the reaction mixture was stopped by adding 6.0 mL of alcohol and 1.0 mL of HCl. Then, 3.0 mL of isooctane was added to extract fatty acid released by olive oil from the reaction mixture. After strongly vortexing for 90 s, 1.0 mL of isooctane layer was taken out and dissolved in 4.0 mL of isooctane and 1.0 mL of copper aeetate monohydrate. Then after strongly vortexing for 90 s, the supernatant was obtained and measured for the inhibition of the lipase activity. Triplicate tests were performed for each sample.

The lipase activity was determined by OD value at 714 nm. Inhibition of the lipase activity was expressed as the percentage decrease in the OD value when PPL was incubated with the test compounds. Lipase inhibitory rate was calculated as follows:

(3) $$\text{Lipase inhibitory rate }(\%)=(1-A_{\text{b}}/A_{\text{a}})\times 100$$

where *A*_a_ represented the lipase activity without any inhibitor, *A*_b_ represented the lipase activity in the presence of hydrolysates.

#### 3.1.4. Assay for α-Amylase Inhibitory Activity

The inhibition assay was performed using the chromogenic DNSA (3,5-dinitrosalicylicacid) method [42]. The total assay mixture composed of 1.0 mL of salivary α-amylase solution and 1.0 mL of sample were incubated at 37 °C for 15 min. After pre-incubation, 1.0 mL of 2% (*v*/*v*) starch solution was added and incubated at 37 °C for 5 min accurately. The reaction was terminated with 2.0 mL DNSA reagent, placed in boiling water bath for 5 min, cooled to room temperature, diluted and the absorbance was measured at 540 nm. α-Amylase inhibitory rate was calculated as follows:

(4) $$\alpha -\text{amylase}\;\text{inhibitory rate }(\%)=(1-A_{\text{b}}/A_{\text{a}})\times 100$$

where *A*_a_ represented the α-amylase activity without any inhibitor, *A*_b_ represented the α-amylase activity in the presence of hydrolysates.

#### 3.1.5. Assay for Degree of Hydrolysis

Degree of hydrolysis (DH) was defined as the percentage of free amino groups cleaved from protein, which was calculated from ratio of α-amino nitrogen (AN) and total nitrogen (TN). The AN was determined by a modified formol titration method [43]. Ten milliliter of FPH was added with an equal amount of distilled water. The mixture was adjusted to pH 7.0 using 0.1 M NaOH. Then 10.0 mL of 38% (*v*/*v*) formaldehyde solution was added into the mixture and titration was continued to the end point at pH 9.5 with 0.2 M standard NaOH solutions. TN was determined by Kjeldahl method [44].

#### 3.1.6. Experimental Design and Statistical Analysis

For the enzymatic reaction, many factors could affect the enzymatic hydrolysis efficiency significantly, such as enzyme specificity, pH, temperature, enzyme dosage, substrate concentration and hydrolysis time [45–48]. The initial assays were based on one-factor design with some different initial pH values, as well as temperatures, enzyme dosages and hydrolysis time. The composition of the model was established from these preliminary assays. The optimal values of the selected variables were obtained by regression analysis on Design-Expert 7.0 (Stat-Ease Inc.: Minneapolis, MN, USA). A four-factor-three-level Box-Behnken design (BBD) was employed in this study, and all 29 of the designed experiments were conducted and performed in triplicate to optimize the four independent variables.

Four main factors namely initial pH (*X*_1_), temperature (*X*_2_), enzyme dosage (*X*_3_), and hydrolysis time (*X*_4_) were chosen as the independent variables. The range and center point values of four independent variables were shown in Table 3. The coded (*χ*) and actual (X) levels of variables in the experimental design were shown in Table 3.

The responses (*Y*) were the PPL inhibitory rate and the α-amylase inhibitory rate. The quadratic model for predicting the optimal point was expressed according to the Equation 5:

(5) $$Y={\text{b}}_{0}+\Sigma {\text{b}}_{i}\chi_{i}+\Sigma {\text{b}}_{ii}{\chi_{i}}^{2}+\Sigma {\text{b}}_{ij}\chi_{i}\chi_{j}$$

where *Y* were the response variables, b_0_, b*_i_*, b*_ii_*, b*_ij_* were the regression coefficient variables, for intercept, linear, quadratic and interaction regression terms, respectively, and *χ_i_* and *χ_j_* were independent variables.

## 4. Conclusions

The hydrolysis condition of fish water-soluble protein by alkaline protease was optimized by using RSM as follows: initial pH 11, temperature 39 °C, enzyme dosage 122 U/mL and hydrolysis time 10 h. To validate the predicted model, the tests were carried out at optimal conditions in triplicate. The PPL and the α-amylase inhibitory rate after optimization could reach 53.21% ± 1.07%, 20.07% ± 0.87% respectively, while predicted values were 54.63% ± 1.75%, 21.22% ± 0.70% respectively. In addition, the manner of PPL inhibition by FPH was analyzed, and the Lineweaver-Burk plots showed noncompetitive inhibition. The *K*_i_ value calculated from the data in Figure 5 was 84.13 mg/mL.

## Figures and Tables

**Table 1:** Box-Behnken design and responses.

RUN	Independent variables				Response (Y_1_) PPL inhibitory rate (%)	Response (Y_2_) α-amylase inhibitory rate (%)

	Initial pH (*χ*_1_)	Temperature (*χ*_2_), °C	Enzyme dosage (*χ*_3_), U/mL	Hydrolysis time (*χ*_4_), h		
1	0	−1	1	0	47.19 ± 1.24	19.44 ± 0.82
2 a	0	0	0	0	53.54 ± 1.15	20.58 ± 0.69
3	0	0	1	1	39.39 ± 0.53	18.22 ± 0.77
4	1	−1	0	0	40.71 ± 1.01	17.92 ± 1.04
5	1	1	0	0	27.58 ± 0.93	18.04 ± 1.12
6 a	0	0	0	0	53.07 ± 1.52	19.24 ± 0.62
7	1	0	0	−1	27.56 ± 1.33	16.61 ± 0.45
8	−1	0	0	1	37.54 ± 1.49	16.92 ± 0.51
9	−1	−1	0	0	43.19 ± 0.58	13.45 ± 0.44
10	1	0	−1	0	29.27 ± 1.02	16.08 ± 0.73
11	0	1	−1	0	27.32 ± 0.75	15.36 ± 0.77
12	0	0	−1	−1	25.63 ± 1.65	11.36 ± 0.36
13	0	1	1	0	40.45 ± 0.87	18.96 ± 0.32
14 a	0	0	0	0	54.15 ± 1.07	20.19 ± 0.54
15	1	0	1	0	42.50 ± 1.44	20.17 ± 0.62
16	−1	1	0	0	35.48 ± 0.95	18.37 ± 0.65
17	0	−1	−1	0	39.32 ± 1.27	14.27 ± 0.49
18	1	0	0	1	37.47 ± 0.82	17.88 ± 0.61
19	0	1	0	−1	31.44 ± 0.98	16.02 ± 0.32
20	−1	0	0	−1	34.52 ± 1.02	11.73 ± 0.47
21	−1	0	−1	0	41.61 ± 1.31	13.98 ± 0.56
22	0	0	−1	1	37.36 ± 0.64	14.29 ± 0.55
23	0	1	0	1	30.39 ± 1.16	15.63 ± 0.69
24	0	−1	0	−1	32.56 ± 1.09	12.08 ± 0.44
25 a	0	0	0	0	54.16 ± 0.85	20.83 ± 0.39
26	0	0	1	−1	47.09 ± 0.72	17.03 ± 0.52
27 a	0	0	0	0	52.01 ± 0.64	21.35 ± 0.42
28	0	−1	0	1	40.53 ± 0.58	16.85 ± 0.31
29	−1	0	1	0	46.82 ± 0.62	18.39 ± 0.37

a center point; PPL, porcine pancreas lipase.

**Table 2:** Results of ANOVA for porcine pancreas lipase (PPL) inhibitory activity and α-amylase inhibitory activity of fish protein hydrolysate (FPH).

Source	The PPL inhibitory rate (%)				The α-amylase inhibitory rate (%)			
	
	Sum of squares	Mean square	*F*-value	*p*-value	Sum of squares	Mean square	*F*-value	*p*-value
Model	2104.13	150.29	48.88	<0.0001 **	209.26	14.95	30.14	<0.0001 **
*χ*_1_	96.73	96.73	31.46	<0.0001 **	16.01	16.01	32.28	<0.0001 **
*χ*_2_	215.39	215.39	70.05	<0.0001 **	5.84	5.84	11.77	0.0041 **
*χ*_3_	330.02	330.02	107.33	<0.0001 **	60.17	60.17	121.34	<0.0001 **
*χ*_4_	47.52	47.52	15.46	0.0015 **	18.65	18.65	37.61	<0.0001 **
*χ*_1_*χ*_2_	7.34	7.34	2.39	0.1445 ns	5.76	5.76	11.62	0.0042 **
*χ*_1_*χ*_3_	16.08	16.08	5.23	0.0383 *	0.026	0.026	0.052	0.8235 ns
*χ*_1_*χ*_4_	11.87	11.87	3.86	0.0696 ns	3.84	3.84	7.75	0.0147 *
*χ*_2_*χ*_3_	6.92	6.92	2.25	0.1559 ns	0.62	0.62	1.24	0.2837 ns
*χ*_2_*χ*_4_	20.34	20.34	6.62	0.0222 *	6.66	6.66	13.42	0.0026 **
*χ*_3_*χ*_4_	94.38	94.38	30.70	<0.0001 **	0.76	0.76	1.53	0.2370 ns
*χ*_1_^2^	409.97	409.97	133.33	<0.0001 **	14.34	14.34	28.92	<0.0001 **
*χ*_2_^2^	520.90	520.90	169.41	<0.0001 **	22.94	22.94	46.27	<0.0001 **
*χ*_3_^2^	195.33	195.33	63.53	<0.0001 **	19.54	19.54	39.41	<0.0001 **
*χ*_4_^2^	756.07	756.07	245.90	<0.0001 **	72.88	72.88	146.98	<0.0001 **
Residual	43.05	3.07	-	-	6.94	0.50	-	-
Lack of fit	39.85	3.98	4.98	0.0678 ns	4.44	0.44	0.71	0.7002 ns
Pure error	3.20	0.80	-	-	2.50	0.63	-	-
Total	2147.17	-	-	-	216.20	-	-	-

*R*^2^ = 0.9800, Adj *R*^2^ = 0.9599, Pre *R*^2^= 0.8908

*R*^2^ = 0.9679, Adj *R*^2^ = 0.9358, Pre *R*^2^ = 0.8636

Notes:

* Significant at *p* < 0.05;

** Significant at *p* < 0.01; ns = not significant.

**Table 3:** Independent variables and their levels used in the RSM experimental design.

Variables	Coded level		

	−1	0	+1
Initial pH (*X*_1_)	10	11	12
Temperature (*X*_2_), °C	35	40	45
Enzyme dosage (*X*_3_), U/mL	60	100	140
Hydrolysis time (*X*_4_), h	7	10	13

*χ*_1_ = (*X*_1_ − 11)/1; *χ*_2_ = (*X*_2_ − 40)/5; *χ*_3_ = (*X*_3_ − 100)/40; *χ*_4_ = (*X*_4_ − 10)/3.
